# Sharenting Syndrome: An Appropriate Use of Social Media?

**DOI:** 10.3390/healthcare11101359

**Published:** 2023-05-09

**Authors:** Ayten Doğan Keskin, Nazan Kaytez, Mustafa Damar, Fatma Elibol, Neriman Aral

**Affiliations:** 1 Department of Child Development, Gülhane Faculty of Health Sciences, University of Health Sciences, Ankara 06010, Turkey; 2 Department of Child Development, Faculty of Health Sciences, Çankırı Karatekin University, Çankırı 18100, Turkey; 3 Department of Child Development, Faculty of Health Sciences, Ankara University, Ankara 06100, Turkey

**Keywords:** child abuse, child neglect, sharenting, social media

## Abstract

Background: The use of social media is on the rise, and posts about anything can be shared these days, whether it be clothes, jewelry, shoes, books, or food and beverages. Some parents even use their children as objects of sharing, and post about their children continuously. Parents who use social media share important moments before and after their children are born on their social network sites accounts. Sharenting refers to the practice of parents, caregivers or relatives sharing information about their children (underage) online, typically on some online platforms. This can include photos, videos, personal stories, and other updates about the child’s life. The study aimed to examine the phenomenon of sharenting syndrome in terms of its potential to cause child abuse and neglect. In addition, the aim of this study is to explore the factors associated with and predicting sharenting syndrome by evaluating it through the lens of child abuse and neglect. Methods: This study was designed with a survey model among quantitative research methods. Data were collected through social network sites with snowball sampling method. The sample consisted of people aged 18 years and over from Turkey (*n* = 427). Results: A total of 86.9% of the participants stated that sharing children’s photos and videos on social media platforms by parents, relatives and caregivers can be evaluated as child neglect and abuse. The variables of “gender” and “the impact of sharing on children” are factors that are associated with determining whether the sharenting syndrome is classified as abuse or not. Gender is a negative predictor of the classification of sharenting on social media as a type of child abuse and neglect. Conclusions: Since the use of social media by people is increasing, there should be measures to protect children from sharenting syndrome.

## 1. Introduction

According to the United Nations Convention on the Rights of the Child, any individual below 18 years of age is considered a child. Unlike in the past, when children were treated as miniature adults, they are now viewed as essential contributors to the future of society. Variables such as the social environment, socio-economic status, care arrangements, access to education and healthcare services, and child and parental health have direct and indirect impacts on children’s lives. Child maltreatment is another factor that can have long-term effects on a child’s life. Child maltreatment refers to the abuse and neglect experienced by children under 18 years of age [1]. Even if the harmful effects of the maltreatment manifest later in life, any act that negatively affects a child’s social–emotional development by an adult responsible for their well-being is considered child abuse and neglect.

Child abuse can take four main forms: sexual, emotional, physical, and economic. Sexual abuse is defined as ‘the abuse of a child or adolescent who has not yet achieved sexual maturity by an adult who uses force, threats or deception to satisfy their sexual desires or needs’ [2]; emotional abuse is defined as “acts that involve behavior or words that have negative effects on a child” [3]; physical abuse is defined as the “non-accidental injury of a child” [4]; and economic abuse is defined as “making children work for financial gain” [5]. There are also more specific acts of child abuse and neglect that do not easily fit into these categories, including Munchausen syndrome by proxy and shaken baby syndrome. Munchausen syndrome by proxy is defined as a parent making up fake symptoms to make it look like their child is sick, and having the child diagnosed and treated for these symptoms [6]. Munchausen syndrome by proxy is a special and severe form of abuse [7]. Shaken baby syndrome, on the other hand, is a form of abuse in which the parent or caregiver shakes the baby or infant by their arms, legs, or body [8], causing severe brain trauma [9]. Experiences of child maltreatment are all related [10] and increase the likelihood of maladjustment and psychopathology [11]. Acts of child abuse can result in permanent physical or mental damage, or even death [12]. Child neglect and abuse can sometimes be perpetrated by the family [13].

The birth of a baby in a family usually brings great joy, and parents may wish to announce the good news and share their happiness in various ways. However, the methods of making such announcements can vary significantly from family to family and culture to culture. Culture can be as influential in a child’s life as their family. In addition to the traditional cultural practices, the mobile age has given rise to a new human culture that has transformed the lifestyles and daily activities of most people [14]. With the increasing role of technology in our lives, the way in which people share news of important developments has also evolved. Parents who use social media platforms share significant moments before and after their children’s birth on their accounts. This practice is commonly referred to as “sharenting,” which involves parents, caregivers, or relatives sharing information about their underage children online, typically on social media platforms such as Instagram, Facebook, Twitter, and others. Sharenting can involve sharing photos, videos, personal stories, and other updates about the child’s daily activities, such as eating, sleeping, bathing, and playing [15]. The word sharenting is derived from the words parenting and sharing [16].

Sharenting is defined as parents sharing content about their children on social media [17] and on the Internet [17,18]. This kind of activity is called sharenting and has been defined by Collins Dictionary as “the practice of a parent to regularly use the social media to communicate a lot of detailed information about their child” (Sharenting, as cited in: Collins Dictionary) [16].

Sharenting syndrome is common among parents [19]. A measurement tool has been developed that can be used to determine the prevalence of sharenting syndrome. Sharenting Evaluation Scale (SES) assesses the degree of sharenting performed by an adult [15].

Sharenting is the practice of using social media to share news and images of children. It carries a negative connotation of parents spending too much time showing the world the happy and fulfilled life of their children instead of really parenting [20]. In this respect, the concept of sharenting carries the negative connotations of disregarding ethics, privacy, and children’s rights [21]. On the other hand, sharenting has positive aspects for parents. The positive aspects of sharenting for parents include the possibility for parents to share the compassion and pride that their children represent in their lives [21]. Social media is not only a place where parents post about their children, but it also serves as a platform where parents can “obtain parenting support and share their parenting experiences”. Some 72 percent of parents who participated in a study reported finding social media to be useful because it makes them feel like they are not alone [22]; some mothers share to validate their parenting and to advise others [23] or to build a network, among other reasons. Parents also use social media to show off their good parenting [21].

When engaging in sharenting behavior, parents may unintentionally expose their children to risks [24]. In a study conducted in Turkey, “social activities carried out with children” was determined to be the most popular subject of parents’ social media posts [25]. According to another study, 42.8 percent of parents’ posts include children. An examination of the accounts of parents who shared their children’s images on Instagram showed that 23.4 percent of parents posted messages containing ads, 13.9 percent posted messages containing locational information, 10.4 percent posted their children’s names, 7.1 percent posted educational and developmental content, and 5 percent posted embarrassing or private content [26].

The potential dangers of sharenting syndrome include the theft of children’s identity information and use of their images on child porn websites [27]. Parents are concerned about the child’s online privacy [28]. According to reports by the National Centre for Missing and Exploited Children, half of the photos shared by child sexual abusers were first posted on social media by parents [29].

Parenthood roles are not well defined in the contemporary world, and the variability and heterogeneity of the parent–child relationship can create uncertainties [17]. Parents set up dedicated accounts for their children for the sharing of pictures and videos or use their own profiles to share such content. Some parents also create YouTube accounts to document their children’s daily activities [30]. Inappropriate or negative comments on children’s images can affect their self-esteem [25], and images can be used by other websites for different purposes.

Representations of children on social networking sites (SNSs) differ between males and females. Male social media celebrities tend to share posts related to “leisure” and “food and drinks”, while female social media celebrities tend to share posts related to “clothing and shoes” and “childcare and health” [31]. The phenomenon of sharenting arises from parents’ desire to be the center of attention [32]. In the pursuit of sharing, parents may infringe on their children’s privacy by presenting them on social media [33]. The exposure of children on social media may be influenced by parents’ own patterns of media use [34]. Parents with higher levels of digital skills are also more likely to engage in sharenting behaviors [35].

The behaviors of sharing and disclosing intimate information about children by their parents on social media platforms is rapidly growing and has become a topic of research for scholars worldwide. Children who become well known on social media may be vulnerable to neglect and abuse due to their developmental characteristics. Child neglect and abuse are not spontaneous situations but are instead caused by people. Similarly, in sharenting syndrome, the child is at risk due to the sharing of the parents, and in some cases may be exposed to neglect and abuse. While sharenting syndrome may not always result in visible harm to the child involved [36], it can lead to problems in social, emotional, or mental development. From this perspective, sharenting syndrome can be exposed a form of child abuse and neglect [36,37].

Emotional neglect, typically characterized by a lack of attention paid to a child’s emotional well-being, can be particularly challenging to identify and assess, particularly when parents or caregivers struggle with addiction issues [38]. Sharenting syndrome has been determined to be strongly associated with technology-based addictions, including internet addiction [39]. The dependency on technology, such as social media and internet addiction, can contribute to families or caregivers emotionally neglecting and abusing their children. The very objects of their addiction, in this case, may be the lives and activities of their children, which can lead to negative consequences for the child’s well-being.

While traditional forms of child abuse inflict direct harm on the child, sharenting can damage the child’s image and pose potential risks to their well-being, both in the present and future. The act of sharenting may result in emotional neglect and abuse, as the caregiver may prioritize the desire to create engaging content for their followers over the child’s needs. In some cases, the caregiver may even coerce the child into repeating certain behaviors or phrases in front of the camera, exploiting the child for entertainment value. Therefore, sharenting can be considered a form of neglect and abuse as it endangers the child during the recording and leaves the child vulnerable to possible harm that may arise from the shared content.

Accordingly, the present study focuses on sharenting syndrome. Various studies have been conducted regarding sharenting [19,20,21,22,23,24,25,26,27,28,29,30,31,32,33,34,35,36,37,38,39,40,41,42,43,44], although none have examined the issue from the perspective of child abuse and neglect. This study aims to examine sharenting syndrome from the perspective of whether it is child abuse and neglect. Hypotheses determined within the scope of the purpose of this research include:

1. The evaluation of sharenting syndrome as child neglect and abuse is associated with specific demographic characteristics, including age, gender, educational level, and socioeconomic status.
2. Parents’ social media use characteristics are associated with the assessment of sharenting syndrome as child neglect and abuse.
3. The evaluation of sharenting syndrome as child neglect and abuse is associated with various forms of child maltreatment, including emotional, physical, and economic neglect and abuse.
4. Some demographic characteristics can predict whether sharenting syndrome can be considered child neglect and abuse.

## 2. Materials and Methods

This study has been designed with survey model among quantitative research methods. Survey model aims to describe a condition as it is [45]. Literature scan has been performed and survey form has been prepared by the researchers. The survey form submitted to expert opinion has been arranged in compliance with the opinions (Table 1).

### 2.1. Sample Size and Procedure

The total population of Turkey is 84,680,273. Internet usage rate of adults (*n* = 63,542,181) is 85% in 2022. In other words, the number of individuals using the internet is 54,010,854. The social media applications that these individuals use the most are WhatsApp, YouTube, and Instagram. The average social media usage is 68.9% (N = 37,213,478) [46].

For calculation of the number of sampled individuals, *n* = (N × t^2^ × p × q)/(d^2^ × (N − 1) + t^2^ × p × q) formula was used. In the formula, t: 1.96, p: 0.50, q: 0.50, d: 0.05 (95% reliability) values were used. Accordingly, the sample size was determined as at least 385 people.

N = (N × t^2^ × p × q)/(d^2^ × (N − 1) + t^2^ × p × q),

*n* = (37,213,478 × (1.96)^2^ × 0.50 × 0.50)/((0.50)^2^ × (1–37,213,478) + (1.96)^2^ × 0.50 × 0.50),

*n* = 385.

In order to reach this sample number, criterion sampling method, one of the non-probability sampling methods, was determined. The criterion was being over 18 years of age and using social media. Individuals for the sample were reached by snowball sampling method.

The survey questions prepared in line with the literature review and expert opinions were entered into Google forms via Google. For sample validity, the research link was delivered to a total of 7 participants, 4 women and 3 men, and a pilot study of the research was conducted. No changes were made to the questions after the pilot study.

Consent on study was obtained from the participants to participate. The data were collected between July 2022 and September 2022. Data were collected through social network sites with snowball sampling method. Participants were included in the study via word of mouth and social network sites. Inclusion criteria required all participants be (a) aged 18 or over and (b) using social networks sites.

The research link was shared on social media accounts (Facebook, Instagram, WhatsApp), and data collection started. During the data collection process, those who filled out the questionnaire were asked to share the survey link with people over the age of 18 and using social media. The data collection process was finalized when the sample size reached a sufficient number. For the accuracy of the sampling frame, it was checked whether the participants in the sample met the criteria determined in the research. Since all participants met the specified criteria, no data were excluded from the sample.

### 2.2. Statistical Analysis

After the prepared survey questions were applied to participants, the obtained data were gathered. The percentages and frequencies of the data gathered from the participants were calculated and reported. In addition, the other opinions stated by the participants were shown with direct quotations. The relation of the variables with sharenting was also evaluated using chi-square test of independence at 95% confidence (*p* ≤ 0.05). Multivariate analysis of categorical variables was binary logistic regression analysis. Variables effective in sharenting syndrome were analyzed by binary logistic regression analysis.

### 2.3. Ethical Approval

The present study was approved by the Ethics Committee of the Çankırı Karatekin University (EC Number: 26/28-06-2022).

### 2.4. Participants

The sample consisted of people aged 18 years and over from Turkey (*n* = 427; 57.6% female). Participants were, on average, 36.2 years old (*SD* = 10.34; age between 18 and 65 years).

## 3. Results

The data obtained within the scope of this study are presented in tables and shown with direct quotations.

Of the participants, 89.2% have an education level of graduate degree, 65.1% are married, 39.6% have a household income of over TRY 12,000, 73.5% are employed, and 63% have children (Table 2).

Of all the participants, 34% have two social media accounts. About one third of the participants with a child (32.8%) do not share the photographs of their children on social media platforms, and the frequency of sharing photographs and videos by those who share is a few days a year (21.1%).

Participants have stated that parents start sharing information about their children on social media platforms when in utero (45.7%); they share a few days a week (39.8%); they share mostly photos on social media platforms (85%); the content of the share is about special days such as birth and birthday (45.9%); and they share for recognition and being known (32.8%). According to the participants, among all other reasons for parents’ sharing about their children on social media platforms, there are statements “addiction”, “unmannerliness”, “ignorance”, “flaunting”, “effort to be popular”, “mental problems”, “boasting of children”, “showing the good sides of children”, “sharing what they believe to be true”, “because they see their children as their most important part”, “because they are happy and they want to share their happiness” (Table 3).

Participants stated that as the result of parents’ sharing on social media platforms about their children, children’s privacy is affected at most (66.3%); it can cause neglect and abuse and cause the visuals of the children to be used on inappropriate sites. The other opinions of participants are: “the sharing group is our close circle, so I don’t think it will cause a problem”, “I think everything will have an effect”, “I think it will have an effect in psychological, social, emotional and private terms”, “Nobody shares bad photos of their own child, the compliments to the child may encourage the child unnecessarily in many subjects”, “I think it changes according to the age of the child”, “Since I share in secure media, I haven’t thought about this”, “It can have an effect in any way”, “The wish to be apparent on social media and the feeling of its necessity develops, causing the possibility of being an addicted individual to increase”, “I don’t think we can talk about a certain situation of affecting or being affected, but if a situation of being affected is concerned, it will cause a negative consequence rather than positive, I think. And this can be in the form of a child’s being too mingled with technology in terms of development and in the form of adopting an existence with lack of productivity and meaning”, “Because the sharing is open to everyone, to public, the content can be used in a way to cause positive or negative results for very different purposes, it has an effects in terms of both social engagement and privacy” (Table 4).

Participants stated that parents most use social media platforms for sharing (52.5%). Other opinions of the participants are “taking a look at soap bubble information for letting themselves go”, “getting away from daily troubles and stress with funny content. For sure, for limited periods”, “for becoming a phenomenon, getting ads, and earning money”, “receiving news and getting information”, “becoming popular”, “it is a kind of a situation that can change for everyone”, “changes according to the person”, “all is suitable”, “spend time”, “addiction”. Participants expressed that mothers (67.2%) use social media platforms more, and parents should obtain permission from their children while sharing about them (92%) (Table 4).

In response to the question “Do you think excessive sharing of children’s photos and videos on social media platforms can be evaluated as child neglect and abuse?”, 86.9% of the participants replied “yes” (Table 4).

All participants were over 18 years of age and used social media. Among the participants who considered sharenting syndrome as child neglect and abuse, 52% were female, 30.9% were between the ages of 36 and 45, 56.2% were married, and 54.3% had children (Table 5).

The variables “gender” and “the ways in which sharenting affects children the most” are factors associated with sharenting syndrome (Table 6). The variables that were not associated with sharenting syndrome were age (x^2^ = 6.044, *p* > 0.05), marital status (x^2^ = 0.215, *p* > 0.05), having children (x^2^ = 0.261, *p* > 0.05), education level (x^2^ = 4.387, *p* > 0.05), income level (x^2^ = 4.084, *p* > 0.05), number of children (x^2^ = 1.644, *p* > 0.05), number of social media accounts (x^2^ = 2.689, *p* > 0.05), status of sharing their children on SNSs (x^2^ = 0.455, *p* > 0.05), frequency of sharing their children on SNSs (x^2^ = 5.086, *p* > 0.05), time of starting to share photos and videos of their child on SNSs (x^2^ = 1.371, *p* > 0.05), content shared on SNSs (x^2^ = 4.369, *p* > 0.05), reason for sharing (x^2^ = 2.020, *p* > 0.05), the purpose of using SNSs (x^2^ = 3.963, *p* > 0.05), the parent who uses SNSs more (x^2^ = 3.434, *p* > 0.05), the status of obtaining permission from the child while sharing on SNSs (x^2^ = 3.516, *p* > 0.05). Based on these findings, Hypotheses 1 and 3 were confirmed.

Sharenting syndrome has been associated with some demographic variables in studies [47,48]. To address this issue, variables related to the demographic characteristics of the participants (such as age, gender) were included in the logistic regression analysis.

Whether sharenting syndrome is considered as abuse and neglect was examined as the dependent variable in a binary logistic regression analysis. The analysis’s significant variables served as the independent variables. The only variable that is effective in evaluating sharenting syndrome as abuse and neglect according to binary logistic regression analysis is gender (Table 7). Based on these findings, Hypotheses 4 was confirmed.

## 4. Discussion

According to this study, approximately one third (32.8%) of participants who have children do not share their children’s photographs on social media platforms. Among those who do share, the frequency of sharing is infrequent, with 21.1% posting only a few times a year. Another study conducted with mothers in Germany determined that 60% of them did not share pictures of their children on social media, while 26% reported sharing such posts less than once a month [23]. In contrast, a study conducted in the United Kingdom with 1002 parents revealed that 71% of parents post five or more pictures of their children on social media every week [49].

The participants of this study reported that parents begin sharing about their children on social media platforms when they are still in utero (45.7%), and they share a few times a week (39.8%). Additionally, most of the content shared is photographs (85%). These findings are consistent with those of the previous literature. For instance, a study conducted in the United Kingdom with 1002 parents determined that 9% of parents shared ultrasound pictures of their unborn babies, 21% set up social media accounts for their children, and 13% took pictures of their children to gain more followers [49]. According to the Child Rescue Coalition based in the US, the average parent posts 1500 pictures of their child on the Internet before the child turns five, and 90% of children appear in pictures or videos posted online before they reach the age of two [50]. An online study was conducted to investigate the digital habits and behaviors of children under the age of nine, involving 6017 parents from ten different countries. The study revealed that 81% of participants had uploaded pictures or videos of their children to the Internet before they turned two years old. Additionally, the study reported that 37% of newborns in the United Kingdom had an online presence from birth, while this figure was 41% for Australia and New Zealand [51].

As parents share images of their children on SNSs, their children begin to develop a digital footprint long before they start walking, and these footprints can follow them into adulthood [41]. Parents intentionally create virtual reality stories that revolve around the lives of their children [40]. The reasons behind parents’ posting of such messages to create these stories can vary. The study participants indicated that parents share these stories for recognition and to become known (32.8%). Additionally, participants stated that parents use social media platforms mostly for sharing (52.5%). A study conducted in Indonesia identified three main reasons why parents share pictures of their children on SNSs: promoting their children, updating their relatives and acquaintances living far away about their children’s development, and displaying pride in their children’s achievements [52]. Parental pride has a long history in parenting [21], and it is a leading emotional factor behind parents’ sharing of images of their children [23]. When parents are proud of their children and their accomplishments, they can showcase their pride through SNSs. In a study examining the reasons for sharing children’s images on social media, one mother stated: “I am proud of my child, and I want to show this to the whole world” [23]. These findings indicate that parents share their children’s images for specific purposes rather than randomly.

In this study, participants expressed that parents’ sharing about their children on social media platforms could have adverse effects, with 66.3% indicating that it could compromise their children’s privacy. Furthermore, it could result in neglect and abuse, and it could lead to children’s images being used on inappropriate sites. These findings are consistent with those of the existing literature.

Parents frequently share details of their children’s daily lives on social media platforms, often without considering the potential impact on their privacy [17]. Sharenting syndrome involves the sharing of not only pictures but also videos, location, as well as information, thoughts, and emotions about the child. In February 2023, France introduced a bill to the Senate aimed at safeguarding children’s privacy and addressing the negative effects of sharing photos and videos of children online. The bill emphasizes the importance of protecting children’s image rights, with both parents being jointly responsible for this task. In cases where parents disagree, a judge may prohibit one of them from sharing images of the child without the other’s consent [53]. This proposed legislation is in line with the findings of this study, which reveal that sharenting can make children vulnerable to abuse.

According to the findings of this study, participants reported that mothers (67.2%) use social media platforms more frequently for sharenting purposes. This finding is consistent with that of a previous study that examined Facebook use among new parents and determined that mothers made more use of the platform during the transition to parenthood than fathers [54]. In some cases, parents even create dedicated social media accounts focused solely on motherhood, which highlights the significance of the parent–child relationship to followers, particularly when a mother identifies herself primarily through her role as a parent [21]. On the contrary, there are studies suggesting that the frequency with which mothers and fathers share their children’s photos on social media is similar [48].

The finding of this study, which suggests that parents should obtain permission from their children before sharing information about them on social media platforms, is in line with the studies in the field. Parents often use social media to share news about their family [21] and may share images of their children without their consent. However, when children become adults, they may feel uncomfortable with the images that their parents have shared. In a study of adolescents, most respondents thought of their parents’ posts and sharenting behaviors as embarrassing and pointless and did not show their approval of sharenting [44]. Similarly, a study in the United Kingdom reported that 32 percent of parents never asked for their children’s consent when sharing their pictures [49]. This highlights the importance of parents seeking their children’s permission before sharing their images on social media.

In a study involving adolescents, it was determined that the respondents generally approved of their parents’ sharenting behaviors, indicating a high level of trust in their parents. However, respondents also reported that discrepancies between their parents’ online image and their own self-image could lead to uncomfortable situations. They stressed the importance of parents obtaining their child’s consent before sharing information about them on social media [43]. Given the developmental characteristics of adolescents who are transitioning from concrete to abstract thinking, questions of identity are particularly important, and peer relationships may become more important than family relationships. Consequently, adolescents’ views may change over time. Although it is recommended that parents obtain their child’s consent before posting their images on social media, it is not possible for babies and infants to make their own decisions or to anticipate the consequences of such decisions. Parents can only obtain consent from school-aged children and older. In a study of Estonian parents and their children aged 9–13 years, discrepancies were detected between the views of parents and children regarding whether the parents had obtained their children’s consent prior to sharing their images [42]. These discrepancies may be due to the child’s age and developmental characteristics, or to differences in the way that parents solicit consent.

All children are vulnerable and open to neglect and abuse. For this reason, the best interests of children should be taken into consideration in every decision and every action taken in relation to children. While children in the 0–6 age group are more dependent on their families, children who have reached the age of puberty may want to decide freely on their own. The age at which children make decisions varies according to the individual. Generally, children over the age of 12 can make decisions. This age may be as low as 7–8 years in some socially developed children. On the other hand, social approach and social environment are also determinative in the formation of ethical rules [55,56,57,58]. The issue of expecting children aged 0–6 to give permission to parents to share their pictures and information about them is confusing. For children under 6 years of age, the best interests of the child should be considered when sharing pictures and information. Children over 6 years of age may be asked for permission to share their pictures and information on social media.

In this study, when asked if excessive sharing of children’s photos and videos on social media platforms could be considered child neglect and abuse, 86.9% of the participants answered affirmatively. The sharing of images of children on social media has also been shown to be a major contributor to images shared on pedophile websites, with nearly half of all such images being obtained from SNSs [59]. In some cases, children’s images and videos shared on social media can result in them becoming micro-celebrities in their communities [60]. However, when parents post with the intention of turning their children into celebrities and continue to share images despite being aware of the potential dangers, sharenting syndrome can be considered a form of child abuse and neglect, akin to Munchausen syndrome by proxy.

In Turkey, a father filed for divorce on the grounds that his spouse exposed their children to abuse by sharing their videos and pictures on social media and having them appear on television shows to become celebrities. The court subsequently issued a ruling prohibiting the mother from sharing children’s videos or pictures on social media [61]. This case demonstrates that courts are beginning to recognize the potential risks of sharing children’s pictures and videos on social media. Similarly, in France, there have been cases of children taking legal action against their parents for sharing their pictures, and the parents have been found guilty once the children turned 18 [62]. These legal cases illustrate that sharing children’s pictures on social media can have long-term consequences and that parents need to be mindful of the potential risks.

Individual children in different countries may experience varying levels of sharenting syndrome, and studies conducted in different countries have reported different results. In one study, parents were discovered to take precautionary measures to respect their children’s privacy after recognizing the risks they face. To avoid harming their children, these parents began engaging in practices such as covering or blurring their faces in pictures and withholding sensitive information, among others [23]. During a debate in Poland about the consequences of oversharing children’s lives, the Children Foundation started a campaign entitled “Pomyśl, zanim wrzucisz!: Think before you upload!” [17]. Some studies also provide recommendations to parents about their social media posts, such as complying with privacy policies and refraining from sharing location or pictures without clothes [41]. However, in sharenting, not only photos and videos are shared, but also data such as the child’s health information [63]. Therefore, it is necessary to evaluate posts from different perspectives, such as health, social–emotional well-being, and privacy. The fact that most of the participants expressed their concerns about sharenting shows that this is an issue worthy of further research and discussion.

In December 2022, the National Centre for Missing and Exploited Children launched the “Take It Down” platform, which is now available worldwide. This free service is designed to help technology companies remove images and videos depicting children under the age of 18 in nude, partially nude, or sexually explicit situations [64]. The objective of such initiatives is to safeguard children from all forms of neglect and abuse.

As a result of a study examining socio-demographic factors that have an impact on sharenting, self-control and internet addiction (*n* = 367, aged between 18 and 61), no socio-demographic factors were defined as predictors of sharenting [39]. However, in another study, it was observed that the frequency of sharing children’s photographs was negatively predicted by the age of the parent [48]. In this study, gender is a negative predictor of the classification of sharenting on social media as a type of child abuse and neglect.

In this study, gender and ways in which children are most affected by sharenting are variables related to whether sharenting syndrome is considered as neglect and abuse. The variables that were not associated with sharing syndrome were age, marital status, having children, education level, income level, number of children, number of social media accounts, status of sharing their children on SNSs, frequency of sharing their children on SNSs, time of starting to share photos and videos of their child on SNSs, content shared on SNSs, reason for sharing, the purpose of using SNSs, the parent who uses SNSs more, the status of obtaining permission from the child while sharing on SNSs.

In one study, psychiatric symptoms of parents and children were determined to be associated with some of the motivations behind sharenting [47]. Another study reported that parental gender, age, and sharing status are related to sharenting, whereas parents’ marriage status, education level, and number of children in the family are not related to sharenting behavior [48]. These findings suggest that certain demographic factors may be related to sharenting behavior, while others may not relate.

While media sharing by parents is often labeled as abusive, it is important to consider the full spectrum of parental motivations and actions. The widespread practice of parents sharing photographs of their children online [36] should not automatically give rise to assumptions of privacy violations or identity manipulation. Rather, each instance of sharing should be evaluated on a case-by-case basis, considering the specific context and potential impact on the child’s well-being [37].

Limitations exist in the current study that must be acknowledged. While the findings may be applicable to cultures such as Turkish, generalization to other cultural contexts should be approached with caution. Sharenting syndrome is a relatively new issue, and the available literature on the topic is not yet comprehensive, particularly in regard to large sample sizes. Further research is needed, including inter-regional comparative studies involving large samples, to improve our understanding of sharenting syndrome.

## 5. Conclusions

Children born in the 21st century have been raised in an environment where social media has become a routine part of their families’ lives. While social media can have both positive and negative effects, access to and harm from the digital world is not evenly distributed among children. One potential harm is child neglect and abuse, which does not occur in isolation. Children are often neglected or abused by others, including their parents, and the same holds true for sharenting syndrome, where parents’ online posts put their children at risk of abuse and neglect.

According to the results of this study, 86.9% of participants believed that sharing photos and videos of children on social media platforms could be considered a form of child neglect and abuse. This article aims to raise awareness among readers, parents, and caregivers that sharenting syndrome can constitute a type of abuse and neglect, and to encourage greater caution in posting about children online.

It is important to recognize that sharenting syndrome can have negative consequences for children’s privacy, autonomy and emotional well-being. Parents who share may unwittingly expose their children to risks such as online harassment, identity theft and cyberbullying. Furthermore, pressure to perform for social media can put children’s emotional and mental health at risk. Sharenting syndrome requires a different intervention than abuse and neglect. Awareness raising, counselling and trainings on sharenting syndrome can protect children. While the child welfare system is already overwhelmed with cases of abuse and neglect, child welfare professionals can play an important role in educating parents about the potential risks of sharing. By encouraging parents to prioritise their children’s best interests when sharing information online and by providing resources and support to help families balance their online and offline lives, child welfare professionals can reduce the potential harm of sharing. It is important to note that sharing should not automatically be equated with child abuse or neglect. Instead, child welfare professionals should work with families to ensure that their online activities do not harm their child’s well-being and privacy. This can be achieved through education, counselling, and support rather than punitive measures that can further burden an already overwhelmed child welfare system.

Official agencies and other organizations also have responsibilities—in addition to parents—of protection of vulnerable groups in society, including children. There is a lack of laws aimed at protecting children from sharenting syndrome, despite the increased use of social media by parents [65]. Therefore, it is important to raise awareness of sharenting syndrome around the world, enlisting the support of the media.

## Figures and Tables

**Table 1:** Delphi method process in the preparation of survey questions.

Purpose	Comments
Round 1	Literature review and preparation of survey questions.
Expert panel selection	Expert 1. Professionals, Master’s degree, nurse, 16–20 years of work experience. Study fields: Health communication, problematic media use. Expert 2. Academics in health sciences, PhD, 26+ years of work experience. Academic study fields: Violence. Expert 3. Professionals, sociologist, Master’s degree, 6–10 years of work experience. Study fields: Health communication, problematic and excessive use of social media, social media addiction. Expert 4. Professionals, PhD, child development specialist, 11–15 years of work experience. Study fields: Child abuse and neglect, behavioral addictions. Expert 5. Academics in health sciences, PhD, 11–15 years of work experience. Academic study fields: Family, child and media.
Send survey questions to expert	The experts’ percentage of compliance: * 80%.
Round 2 Send survey questions to expert	Suggestions and corrections were made in the questionnaire. The experts’ percentage of compliance: 88%.
	Delphi method process completed.

* Percentage of compliance = consensus (total number of questions-difference of opinion)/total number of questions × 100.

**Table 2:** Demographical information of participants.

	F	%
Ages		
18–25 years	78	18.3
26–35 years	122	28.6
36–45 years	148	34.7
46 years and above	79	18.5
Education level		
Graduate	381	89.2
High school	37	8.7
Primary school	9	2.1
Marital status		
Married	278	65.1
Single	139	32.6
Other (Divorced, widow)	10	2.3
Household income		
Over TRY 12,000	169	39.6
Between TRY 8001 and 12,000	135	31.6
Between subsistence wage and TRY 8000	80	18.7
Less than subsistence wage	43	10.1
Employment status		
Employed	314	73.5
Unemployed	113	26.5
Number of children		
Two	126	29.5
One	92	21.5
Three	40	9.4
Four	11	2.6

**Table 3:** Thoughts of participants about social media use of parents who use social media.

	F	%
When do you think parents start to share their children’s photos and videos on social media platforms?		
In utero	195	45.7
With birth	121	28.3
After birth—before 2 years of age	40	9.4
After 2 years of age	71	16.6
How often do you think parents share their children’s photos and videos?		
Every day	86	20.1
A few days a week	170	39.8
A few days a month	69	16.2
A few days a year	63	14.8
Never	39	9.1
What do you think parents share about their children on social media platforms?		
Photographs (photos with the child, photos with only the child)	363	85
Videos (videos with the child, videos with only the child)	38	8.9
Information of the child (name, school, location, etc.)	3	0.7
Feelings and thoughts about the child (joy, pride, happiness, etc.)	15	3.5
Other (both photographs and videos, everything)	8	1.6
What type of content do you think parents share about their children on social media platforms?		
Birth, special times such as birthdays, content about special days	196	45.9
Content about eating or cooking	7	1.6
Content about playing games	25	5.9
Everything	195	45.7
Other (arts activities, drawings, clothes)	4	0.8
What do you think is the basis of parents’ sharing about their children on social media platforms?		
Forming an archive about the child	85	19.9
Socialization	79	18.5
Informing and recommending to others	24	5.6
Recognition, being known	140	32.8
Confirming parenting	70	16.4
Other	29	5.8

**Table 4:** Participants’ thoughts about social media usage of parents who use social media and its effects on their children.

	F	%
In which way do you think sharing children’s photos and videos on social media platforms affects children at most?		
In an emotional way (words or comments that affect/will affect the child negatively, etc.)	87	20.4
In an economic way (using for advertisements or gaining income, etc.)	9	2.1
Affects privacy (can cause negligence or abuse, or can cause the visuals of child to be used in inappropriate sites, etc.)	283	66.3
No effect; other opinions	48	10.8
For what purpose do you think parents use social media platforms most?		
Access to useful information	32	7.5
Communication	45	10.5
Socialization	115	26.9
Sharing	224	52.5
Other opinions	11	2.2
Do you think mothers or fathers use social media platforms at most?		
Mothers	287	67.2
Fathers	18	4.2
Both	122	28.6
Do you think parents must obtain permission from their children while sharing their photos and videos on social media platforms?		
Yes	393	92
No	34	8
Do you think excessive sharing of photos and videos of children on social media platforms can be evaluated within the context of child neglect and abuse?		
Yes	371	86.9
No	56	13.1

**Table 5:** Characteristics of the participants who stated that sharenting syndrome can be considered as child neglect and abuse.

Variables		Do You Think Excessive Sharing of Photos and Videos of Children on Social Media Platforms Can Be Evaluated within the Context of Child Neglect and Abuse?		
		Yes (%)	No (%)	Total (%)
Gender	Female	222 (52%)	24 (5.6%)	246 (57.6%)
	Male	149 (34.9%)	32 (7.5%)	181 (42.4%)
Ages	18–25 years	69 (16.2%)	9 (2.1%)	78 (18.3%)
	26–35 years	108 (25.3%)	14 (3.3%)	122 (28.6%)
	36–45 years	132 (30.9%)	16 (3.7%)	148 (34.7%)
	46 years and above	62 (14.5%)	17 (4%)	79 (18.5%)
Marital Status	Married	240 (56.2%)	38 (8.9%)	278 (65.1%)
	Single and Other (Divorced, widow)	131 (30.7%)	18 (4.2%)	149 (34.9%)
Having a child	Yes	232 (54.3%)	37 (8.7%)	269 (63%)
	No	139 (32.6%)	19 (4.4%)	158 (37%)
	Total	371 (86.9%)	56 (13.1%)	427 (100%)

**Table 6:** Variables associated with whether the evaluation of sharenting syndrome is within the scope of neglect and abuse.

Variables		N (%)	Sharenting Is Child Neglect and Abuse	Sharenting Is Not Child Neglect and Abuse	χ^2^	*p*-Value
**Gender**	Female	246 (57.6%)	222 (52%)	24 (5.6%)	5.745	0.017 *
	Male	181 (42.4%)	149 (34.9%)	32 (7.5%)		
**In which way sharenting affects children at most**	In an emotional way	87 (20.4%)	73 (17.1%)	14 (3.3%)	8.701	0.034 *
	In an economic way	9 (2.1%)	8 (1.9%)	1 (0.2%)		
	Affects privacy	283 (66.3%)	254 (59.5%)	29 (6.8%)		
	Do not affect and other	48 (11.2%)	36 (8.4%)	12 (2.8%)		

* Chi-square test of independence at 95% confidence (*p* ≤ 0.05).

**Table 7:** Results of binary logistic regression analysis results for predicting sharenting syndrome.

Independent Variable	B	S.E.	Exp(B)	*p*-Value
Gender				
Female (Ref.)				
Male	−0.711	0.333	0.491	0.033 *
Age				
18–25 years (Ref.)				
26–35 years	−0.690	0.712	0.502	0.333
36–45 years	−0.699	0.495	0.497	0.158
46 years and above	−0.618	0.406	0.539	0.128
Education level				
Primary school (Ref.)				
High school	0.552	0.892	1.737	0.536
Graduate	−1.906	1.041	0.149	0.067
Household income				
Less than subsistence wage level (Ref.)				
Between subsistence wage level and TRY 8000	−0.737	0.651	0.479	0.257
Between TRY 8001 and 12,000	−0.494	0.493	0.610	0.317
Over TRY 12,000	−0.646	0.363	0.524	0.075
Marital status				
Married (Ref.)				
Single and Other	−0.021	0.556	0.979	0.970
Having a child				
Yes (Ref.)				
No	−0.172	0.609	0.842	0.778
Employment status				
Employed (Ref.)				
Unemployed	−0.638	0.465	0.528	0.170

Note: * (*p* ≤ 0.05).
